# NIPS, a 3D network-integrated predictor of deleterious protein SAPs, and its application in cancer prognosis

**DOI:** 10.1038/s41598-018-24286-2

**Published:** 2018-04-16

**Authors:** Bo Wang, Jing Li, Xi Cheng, Qiao Zhou, Jingxu Yang, Menghuan Zhang, Haifeng Chen, Jing Li

**Affiliations:** Department of Bioinformatics and Biostatistics, School of Life Sciences and Biotechnology, Shanghai Jiao Tong University, Shanghai, China

## Abstract

Identifying deleterious mutations remains a challenge in cancer genome sequencing projects, reflecting the vast number of candidate mutations per tumour and the existence of interpatient heterogeneity. Based on a 3D protein interaction network profiled via large-scale cross-linking mass spectrometry, we propose a weighted average formula involving the combination of three types of information into a ‘meta-score’. We assume that a single amino acid polymorphism (SAP) may have a deleterious effect if the mutation rarely occurs naturally during evolution, if it inhibits binding between a pair of interacting proteins when located at their interface, or if it plays an important role in a protein interaction (PPI) network. Cross-validation indicated that this new method presents an AUC value of 0.93 and outperforms other widely used tools. The application of this method to the CPTAC colorectal cancer dataset enabled the accurate identification of validated deleterious mutations and yielded insights into their potential pathogenesis. Survival analysis showed that the accumulation of deleterious SAPs is significantly associated with a poor prognosis. The new method provides an alternative method to identifying and ranking deleterious cancer SAPs based on a 3D PPI network and will contribute to the understanding of pathogenesis and the discovery of prognostic biomarkers.

## Introduction

The accumulation of DNA mutations can cause cancer^1^, particularly when these mutations occur in coding regions and lead to single amino acid substitutions^2,3^. Recent advances in high-throughput sequencing technologies have promoted the identification of many somatic mutations by ongoing initiatives, such as The Cancer Genome Atlas (TCGA; http://cancergenome.nih.gov) and the International Cancer Genome Consortium (ICGC; https://dcc.icgc.org)^4,5^. These initiatives have shown that cancer genomes often contain hundreds or thousands of mutations; however, not all of these mutations appear to play a functional role in tumour development. In fact, among the 2,000,000 coding mutations described in COSMIC (version 70), most mutations have no effect on disease development^6^, and only a few of these changes are closely associated with or lead to cancer. These changes are referred to as deleterious mutations or, at the protein level, deleterious single amino acid polymorphisms (SAPs)^7,8^. Deleterious mutations in cancers are closely associated with early diagnosis, personal therapy and prognostic prediction^9–11^.

Identifying deleterious SAPs in a cohort of tumours is a key challenge in cancer omics studies. Many strategies for predicting the effects of SAPs on protein function have been developed. Among these strategies, SIFT (Sorting Intolerant From Tolerant) is a reliable and widely used method for predicting deleterious or tolerated SAPs^3,12^. LogRE (Log R Pfam E-value) predicts the effect of a SAP by evaluating the sequences of Pfam domains between wild type and mutant alleles^13,14^. In addition to the sequence information, protein structural information is also helpful. PolyPhen-2 is a prominent tool that uses both sequence- and structure-based features in a naïve Bayes classification^15,16^. As a cancer-specific tool, CHASM (cancer-specific high-throughput annotation of somatic mutations) is a major machine-learning approach employing a random forest algorithm^17^ and was trained using 49 predictive features, including conservation exon information, UniProt annotations and the frequency of missense changes in the COSMIC database^6,18^. These tools primarily rely on the characteristics of and evolutionary information for an individual protein sequence and ignore the effect of the mutation on protein interactions and topology in the protein-protein interaction (PPI) network. Indeed, cellular processes and biological functions are rarely attributed to the activity of a single protein. Instead, proteins act in functional modules, such as macromolecular complexes or signal transduction networks^19,20^. Since aberrant PPIs can have drastic effects on biochemical activities that are essential to the homeostasis, growth, and proliferation of cells, leading to various human diseases, determination of the proximity of a mutation to known disease-related proteins in a PPI network can aid in the detection of important proteins or deleterious SAPs^21^. For example, the loss of key novel interactors that promote ΔF508 CFTR channel function in primary cystic fibrosis epithelia and proteins critical for CFTR biogenesis was recently identified by identifying the CFTR mutation-specific interactome^22^. In addition, Yu *et al*. predicted a 3D protein interactome network with structural resolution and found that disease-associated mutations are significantly enriched at protein interaction interfaces^23,24^. Notably, cross-linking mass spectrometry has recently emerged as a powerful technology for identifying both the interactions and interaction interfaces between proteins on a large scale *in vivo*^25,26^. Several follow-up studies have profiled thousands of *in vivo* PPIs with interface structures in living human cells using the most recent cross-linking technologies^20,27–29^. These analyses offer the alluring opportunity to study the relationships between protein functions and interaction structures.

Here, we describe a new method, referred to as NIPS, that integrates 3D interface interactions, network topology and information on sequence evolution to determine which mutations identified in cancer genomes are likely to be deleterious. The cross-validation revealed that as an integrative method, NIPS shows better performance than methods based on individual information, also outperforms other widely used tools. The area under the receiver operating characteristic (ROC) curve (AUC) of NIPS reached 0.93, indicating that this method is highly accurate. We applied this method to 796 somatic SAPs previously detected in 95 colorectal cancer samples using RNA-Seq and mass spectrometry^30^. For some deleterious SAPs predicted using NIPS, we conducted a network-based analysis and molecular dynamics simulation of the interaction structure. In addition, we used the predicted deleterious SAPs to classify 86 colorectal samples. The results showed that accumulating deleterious SAPs were significantly associated with a poor survival rate, while the neutral SAPs showed no correlation. These results confirm the reliability of NIPS and increase the current understanding of the pathogenesis of known deleterious SAPs. Users can discover new deleterious SAPs and markers related to the prognosis of cancer using NIPS.

## Results

### A 3D network-integrated method for prediction of deleterious SAPs

**Figure 1:**
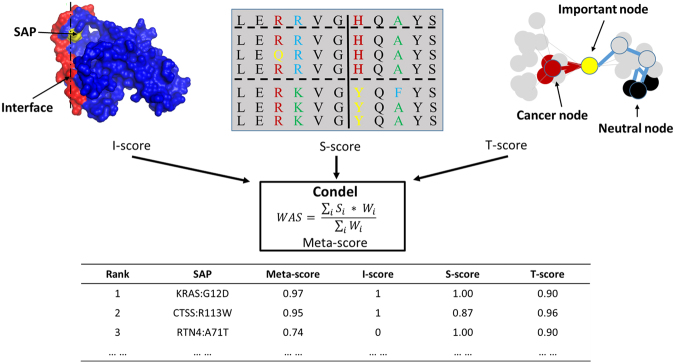
The NIPS workflow. I-score: two interacting proteins (red and blue) form a complex. The mutation (yellow) is localized with respect to the interface. If the mutation is at the interface, the I-score is 1; otherwise, the score is 0. The T-score is a measure of the proximity of a mutant node to a known cancer node in the protein-protein interaction network compared with the distance to a neutral node. The S-score is derived from the original score predicted by SIFT. The S-score is equal to 1 - SIFT score. A weighted average formula, Condel, was used to integrate these three scores into the ‘meta-score’. All SAPs were ranked according to their meta-scores.

To generate the 3D network-integrated risk predictor of somatic SAPs (NIPS) tool, we integrated a 3D PPI network interface, network topology, and information on sequence evolution. First, we identified SAPs located at the interface between pairs of interacting proteins identified based on cross-linking experiments and INstruct data, as these mutations may disrupt protein interactions (generating the I-score). Next, the ratio of the average shortest paths to cancer nodes and non-cancer nodes in the protein-protein interaction network (the T-score) was used to measure the proximity of a mutated node to known cancer nodes. This score is based on the assumption that when a mutated node is closer to a known cancer-related node in the network, the more likely it is that the mutation is deleterious. We also used the SIFT method to evaluate the potential deleteriousness of mutations using protein sequence evolution information (to generate the S-score). Finally, we combined these three normalized individual scores (0 to 1) into a weighted average ‘meta-score’ to evaluate the risk of a SAP. The workflow of the development of our network-based predictor is shown in Fig. 1.

**Figure 2:**
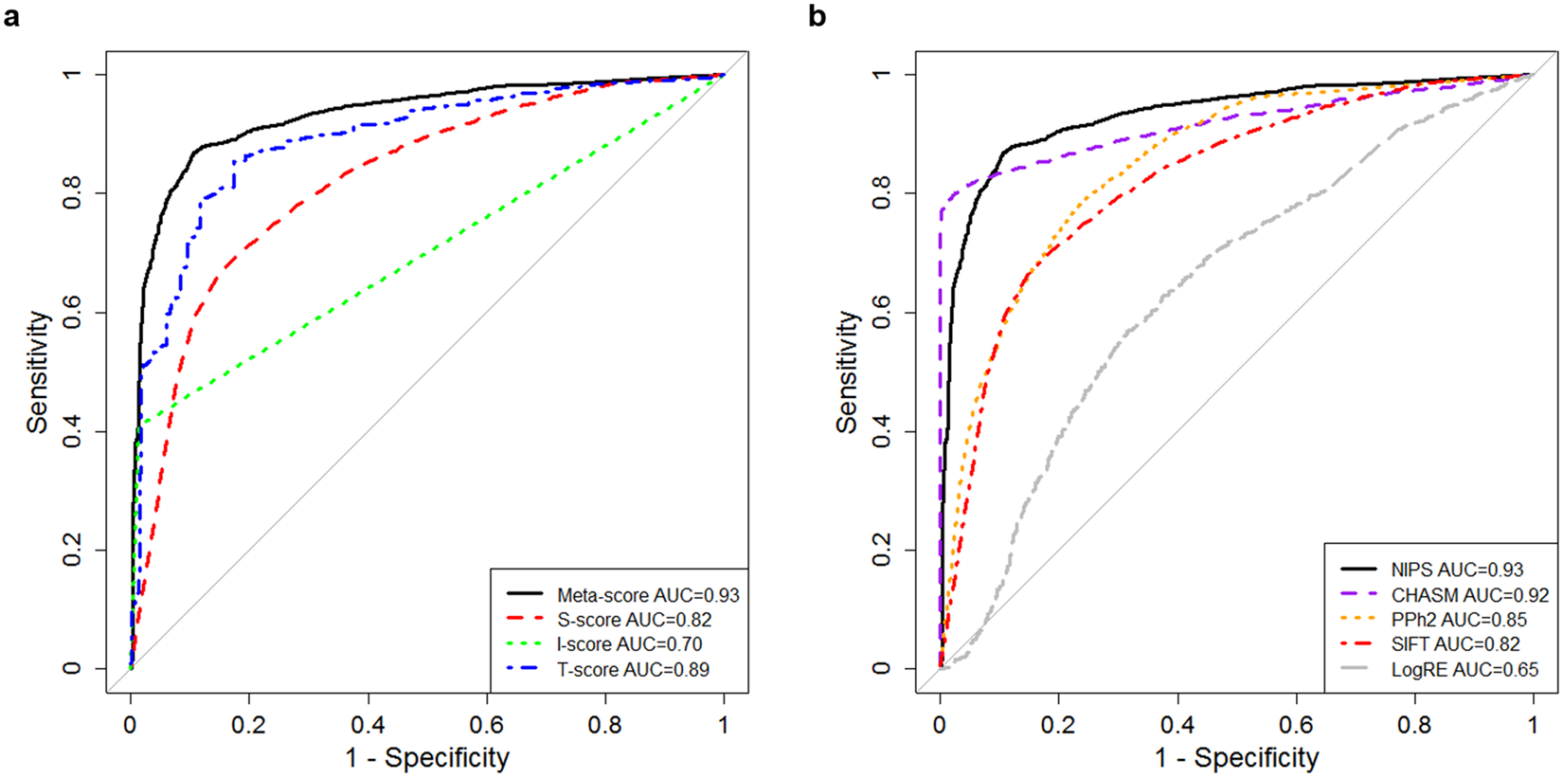
ROC curves across scores and tools. (**a)** ROC curves for the meta-score, S-score, I-score and T-score in NIPS using the training datasets. The performance of the meta-score is best. (**b)** ROC curves for NIPS, SIFT, Condel, PolyPhen-2 (PPh2), LogRE and CHASM. The AUC values are attached, and NIPS shows the best performance.

Using the training dataset described in the Methods section, we evaluated the performance of the meta-score of NIPS in cross-validation and the S-score, T-score and I-score individually (Fig. 2a). Although the AUC value of the T-score was higher than that of the other two scores, the meta-score performed significantly better than the T-score (DeLong’s test for two ROC curves using the *pROC* package for R^31^, the p-value is 1.5e-13). This finding suggests that the integration of three different types of data sources can improve the accuracy of the identification of deleterious SAPs. We further performed comparisons with widely used tools, including SIFT (S-score), LogRE, PolyPhen-2 and CHASM, using the same test dataset. As shown in Fig. 2b, NIPS outperformed the other tools, achieving an AUC of 0.93, which was the highest AUC value obtained. In Table 1, we list the prediction accuracies, sensitivities, specificities, AUC values and Matthew’s correlation coefficients (MCC) across the evaluated tools. NIPS achieved the highest accuracy (88.6%), MCC (0.73) and sensitivity (86.7%), which were all slightly better than the values for CHASM. CHASM was superior in terms of specificity.

**Table 1:** Prediction accuracies, sensitivities, specificities, AUC values and Matthew’s correlation coefficients (MCC) between the evaluated tools.

Tool	Accuracy	Sensitivity	Specificity	AUC	MCC
NIPS	88.6%	86.7%	89.4%	0.93	0.73
CHASM	87.1%	74.6%	92.2%	0.92	0.73
PPh2	71.6%	69.2%	72.5%	0.85	0.50
SIFT	73.6%	66.9%	76.1%	0.82	0.47
LogRE	24.5%	63.7%	8.8%	0.65	0.07

### Identifying deleterious SAPs in the CPTAC colorectal cancer dataset

We applied NIPS to the colorectal cancer proteome dataset from CPTAC. Among the 795 candidate SAPs identified from 95 colorectal tumour samples^30^, we identified 85 deleterious SAPs with a false positive rate of less than 10%, for which the meta-score was above the cutoff score of 0.9. Among these deleterious SAPs identified by NIPS, only 21 were predicted by SIFT too. Among the deleterious SAPs predicted exclusively using the NIPS method, many of which have been reported in other tumour type, or reported for their association with the progression of colorectal cancer, pancreatic cancer and gastric cancer, such as G12S in KRAS, W383G in CTNNB1, R517K in COL4A2, and V44I in LASP1^32–39^.

**Figure 3:**
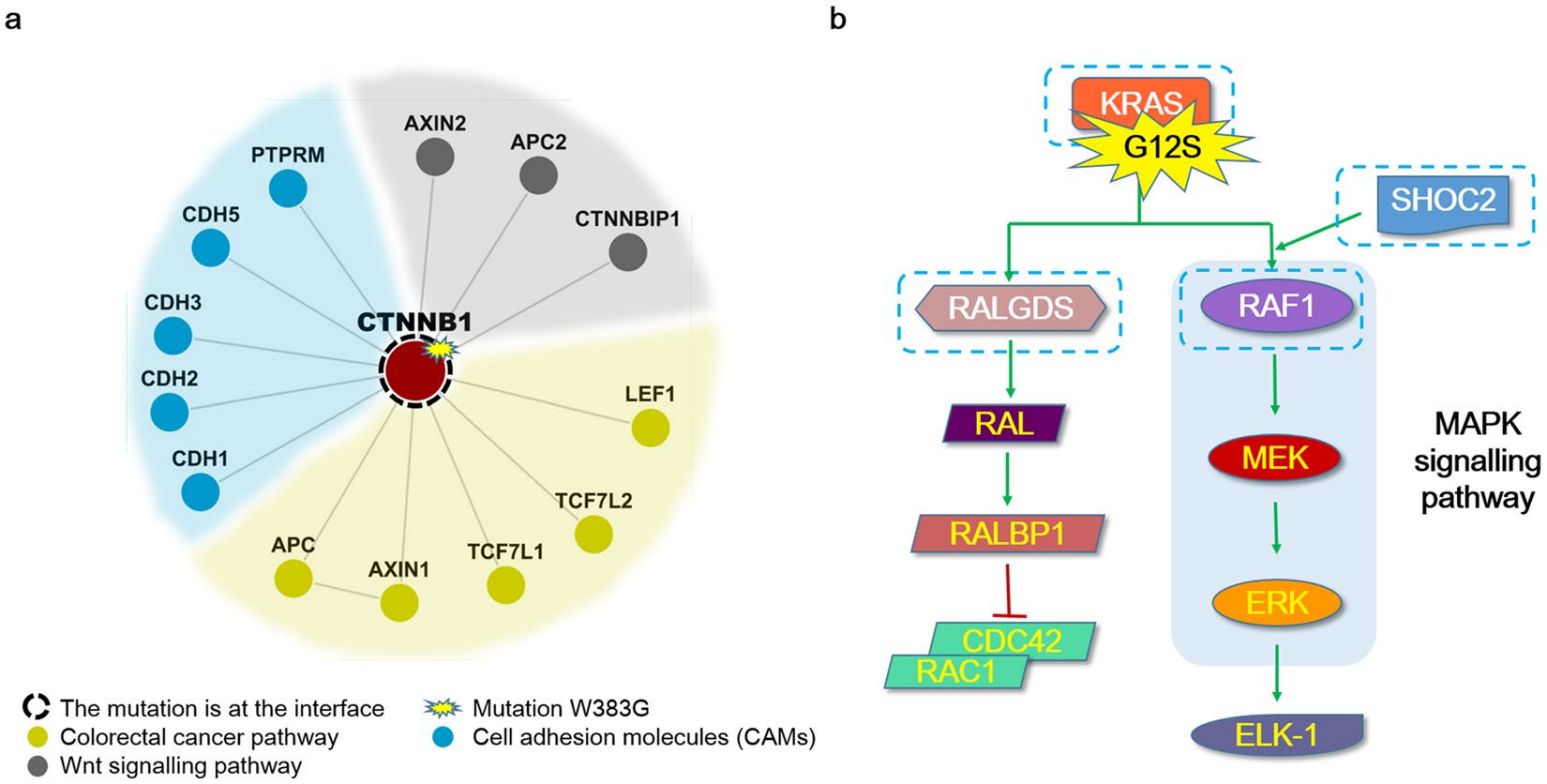
Downstream interactors affected by deleterious SAPs in CTNNB1 and KRAS. (**a)** Neighbours of CTNNB1 mutants, classified into three functional groups: the colorectal cancer pathway, cell adhesion molecules (CAMs), and the Wnt signalling pathway. (**b)** KRAS and its interaction neighbours RALGDS, SHOC2, and RAF1 (dashed boxes) in the Ras signalling pathway. The deleterious SAP G12S is located at the interaction interface.

The SAPs CTNNB1 W383G, KRAS G12D and G12S occur in the known oncogenes^7^. NIPS classified these mutations as deleterious, reflecting their high I-scores. Based on a previous study^22^, we hypothesized that mutations at the interface could result in impairment or loss of the corresponding interactome. Thus, we investigated how these deleterious SAPs affect the CTNNB1 and KRAS interactome. As shown in Fig. 3a, the W383G mutation lies at the interface between CTNNB1 and its 13 neighbours in the 3D interaction network, and affects their interactome. Functional analysis revealed these neighbours enriched in cell adhesion molecules as well as the colorectal cancer and Wnt signalling pathways. Therefore, this SAP may altering these three pathways by altering the bonds and interaction structure between proteins. A new study found that mutation W383G in CTNNB1 occurred together with recurrence of prostate cancer^40^. As an interaction pair in colorectal cancer pathway and Wnt signalling pathway, genomic alterations in the pair of APC and β-catenin (CTNNB1) significantly associate with reductions in DFS (disease-free survival) in patients with prostate cancer, It’s interesting that mutations in APC are mutually exclusive from those occurring in β-catenin in both colon cancer and prostate cancer^40,41^. KRAS and its three interaction partners (Fig. 3b), RALGDS, SHOC2, and RAF1, play key roles in the Ras signalling pathway, which leads to cell apoptosis pathways. The G12S and G12D mutations are located at the interface between KRAS and these partners, indicating that these SAPs may affect cell apoptosis-related functions via interrupting the connections between KRAS and its downstream elements, leading to cancer. The G12D mutation results in an amino acid substitution at position 12 in KRAS, from a glycine (G) to an aspartic acid (D), which is classified as deleterious by both SIFT and NIPS. G12D mutation is a known driver mutation and drug target in cancer^42,43^, of which frequency among KRAS-mutated colorectal cancers is 33.5–34.4%^6^. Another mutation at position 12 G12S shows much lower frequency among KRAS-mutated colorectal cancers (4.9–5.7%)^6^. It was identified as a deleterious mutation by NIPS due to high I-score while being classified as a neutral one using SIFT. More recently, Ortiz-Cuaran *et al*. reported that KRAS G12S mutation is significantly related to acquired drug resistance in cancer. Furthermore, introduction of KRAS G12S resulted in increased KRAS expression and sustained ERK phosphorylation under treatment with drug AZD9291, which provide clinical evidence for a possible role of MAPK pathway activation in the context of acquired resistance to third-generation EGFR inhibitors^44^. In our analysis, the NIPS identified the deleteriousness of KRAS G12S mutation accurately. More importantly, it provided insight to its possible mechanism and the link between the mutation and the downstream MAPK pathway (Fig. 3b). The full list of the deleterious mutations identified in human colorectal cancer samples by NIPS is provided in Supplementary Table S1.

### Protein interaction structure and topology attacked by deleterious SAPs

**Figure 4:**
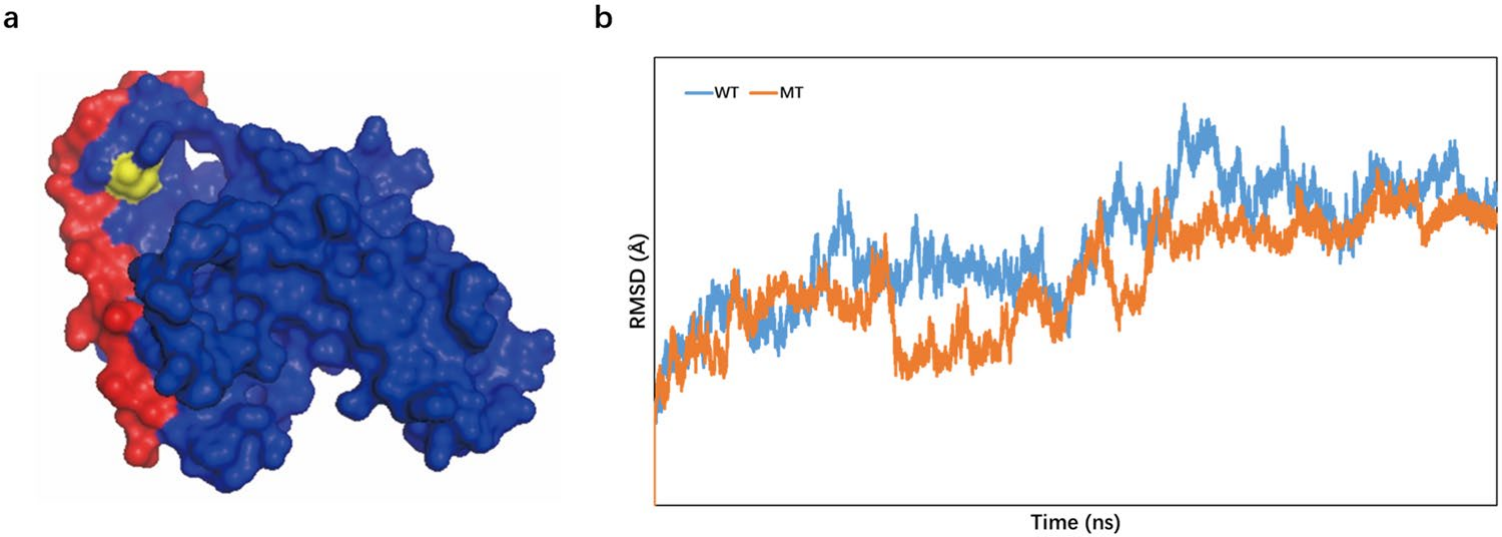
Molecular dynamics simulation of the HLA-DQA1 and HCRT complex. (**a)** 3D structure of the HLA-DQA1 (blue) and HCRT (red) complex. The yellow site corresponds to the amino acid HLA-DQA1 M99. This image was built using PyMol software^79^. (**b)** RMSD of atomic positions between HLA-DQA1 and HCRT. The blue line represents the wild type, and the red line represents the M99V mutation (mutant type).

As described above, SAPs at the interface may weaken or disrupt protein interactions and then affect the function of pairs of interacting proteins. Here, molecular dynamics simulations were performed to illustrate changes in protein structure resulting from SAPs. The expression of orexin-A (HCRT) regulates the onset and progression of prostate cancer^45^. Its physical interaction partner HLA-DQA1 plays a central role in the immune system and is associated with an increased risk of drug-induced hepatotoxicity in patients with breast cancer^46–48^. Although the biological role of the interaction between HLA-DQA1 and HCRT in cancer remained unknown until recently, the HLA-DQA1 M99V SAP at the interaction interface between these two important proteins may affect binding to its partner (Fig. 4a), which was predicted as deleterious but was classified as ‘tolerated’ (non-deleterious) via the SIFT method. To verify this observation, we applied molecular dynamics simulations to calculate the binding free energy of these two interacting proteins with and without the SAP. The protein structure (PDB id: 1UVQ) can illustrate how HLA-DQA1 interacts with HCRT^49^. The results indicated that the presence of this mutation leads to a change in binding free energy between these proteins from −65.16 kcal/mol (wild type) to −42.26 kcal/mol (mutant) (Fig. 4b). The root-mean-square deviation (RMSD) of atomic positions revealed the relative distance between the proteins^50^; both lines were stable, suggesting that 50 ns is sufficient for molecular dynamics.

**Figure 5:**
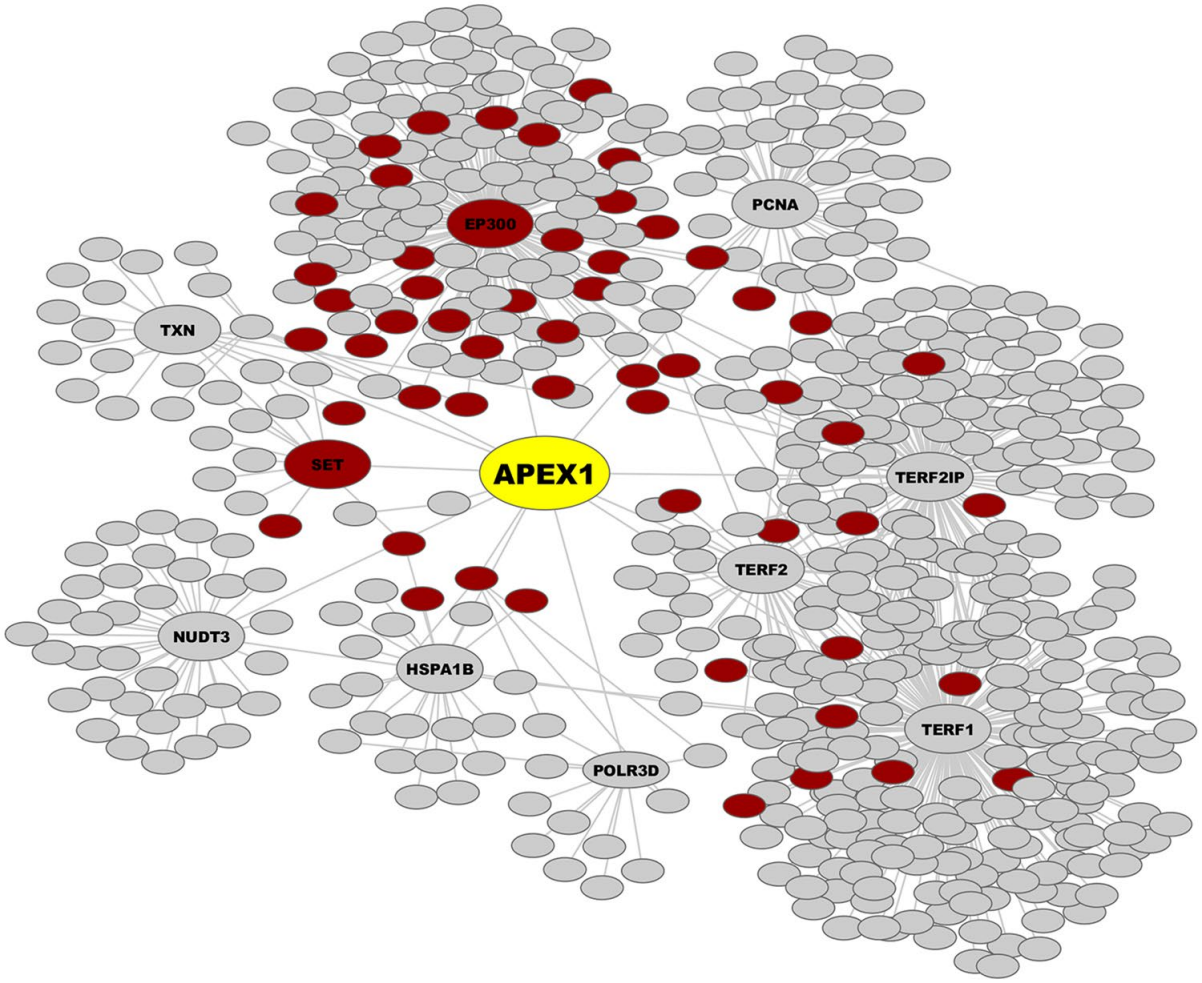
Sub-network topology of APEX1 and its close neighbours. Shortest paths of APEX1 to its neighbours with ≤2 steps are displayed. Red nodes correspond to cancer-related proteins.

Some SAPs were classified as deleterious by NIPS because of high T-scores. For example, the D148E mutation of APEX1 received an S-score of 0 and a T-score of 0.93. In addition to its role in DNA repair, APEX1 (apurinic/apyrimidinic endonuclease 1; also known as APE1) is a transcriptional regulator^51^. A meta-analysis of 15 studies involving 4,932 lung cancer patients and 6,555 cancer-free controls found that in an Asian population, carriers of APEX1 D148E exhibited an increased risk of developing lung cancer^52^. Moreover, the presence of this mutation increased the risk of gastric cancer and affected the survival of patients with urothelial carcinoma of the bladder in a Chinese population^53^. The high T-score of the D148E mutation in APEX1 suggests that the mutant protein is closer to cancer-related nodes than neutral nodes in the protein interaction network. We randomly sampled 1,000 nodes in the background network and calculated the shortest path from APEX1 to each node. We found that the average length of shortest paths of APEX1 to cancer-related nodes was 3.6, which was less than the average distance to non-cancer-related nodes of 4 (p-value = 7.16e-05, Wilcoxon rank sum test). All of the interaction partners of APEX1 within a maximum of two steps are displayed in Fig. 5. Cancer-related nodes were significantly enriched in this sub-network compared with the whole background network (hypergeometric test p-value = 6.48e-7), suggesting that if an important node (protein) is mutated, the overall topology of the network might be compromised, and the efficiency of the signal transmission will be affected.

### Accumulation of deleterious SAPs and poor prognosis

**Figure 6:**
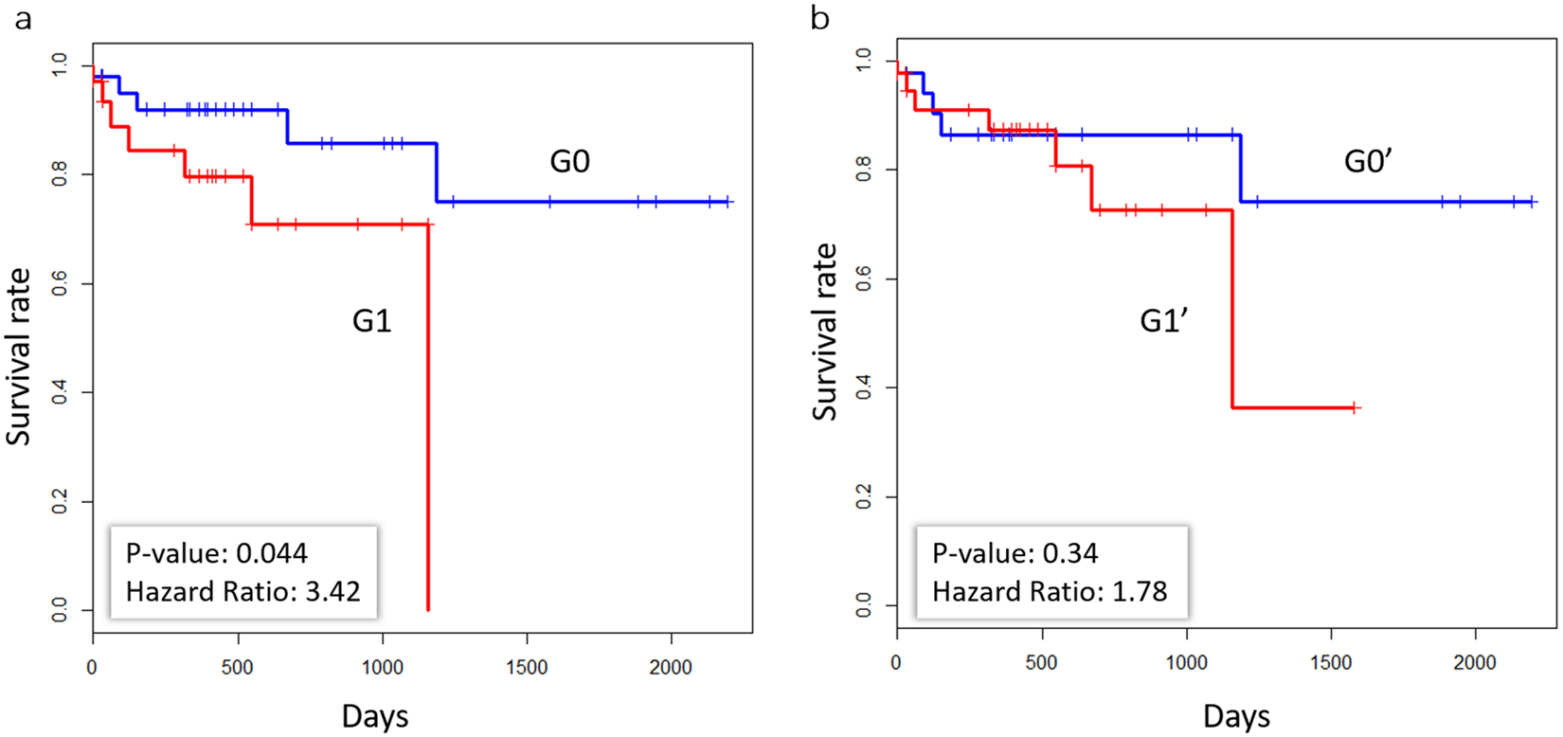
Kaplan-Meier plot of overall survival stratified by the sum of meta-scores. Survival curves are shown on the y-axis versus survival time in days on the x-axis. (**a)** The 84 colon cancer samples were classified into two groups, G1 and G2, using the sum of the meta-scores of the top 30 deleterious SAPs. The G1 group (red, above the mean) shows a much higher risk than the G2 group (blue, below the mean), with a hazard ratio of 3.42 (log-rank test p-value = 0.044). (**b)** Survival comparison between the groups classified by the meta-scores of all SAPs detected in the samples. The hazard ratio between the G1′ group (red, above the mean) and the G0′ group (blue, below the mean) is 1.70 (log-rank test p-value = 0.38).

The availability of the TCGA survival data enabled the investigation of the relationship between the accumulation of the deleterious SAPs and the overall survival of patients. We investigated the correlation between the accumulation of deleterious SAPs and survival in 84 of the 95 colorectal tumour samples with available survival information. Based on the summation of the meta-scores of the top 30 SAPs in each sample, these patients were classified into two groups: G1 (high-risk group), in which the sum of the meta-scores of each sample was higher than the mean value 2.99, and G0 (low-risk group), in which the sum of the meta-scores of each sample was below the mean value. As shown in Fig. 6a, the survival of the high-risk group was much worse than that of the G0 group, and the hazard ratio obtained using the Cox proportional hazards regression model was 3.42 (log-rank test p-value = 0.044). For comparison (Fig. 6b), the survival rates of the two groups (above or below the mean value) were not different (hazard ratio = 1.70, log-rank test p-value = 0.38) when all of the SAPs were used, suggesting that the accumulation of the deleterious SAPs was strongly associated with patient survival.

### NIPS website

The results and data obtained in this study are available for download at lilab.life.sjtu.edu.cn:8080/nips. Users can search all of the known SAPs annotated in the CanProVar database, which stores single amino acid alterations in the human cancer proteome^8,54^ and ranks SAPs from the local candidate list. Based on the S-score, I-score, T-score, and meta-score, any new SAP identified via human cancer genome sequencing can be evaluated and ranked using the NIPS server. The 3D PPI network and the training datasets can also be downloaded.

## Discussion

In the present study, we developed an integrative approach referred to as NIPS, employing a meta-score to evaluate the risk of SAPs computationally and identify deleterious SAPs in cancer by combing information on PPI 3D structure (I-score), network topology (T-score), and sequence conservation (S-score). NIPS can be used to identify new deleterious SAPs in cancer genome or proteome data, which would be helpful for early detection or target-therapy. For instance, the 70 proteins containing deleterious SAPs identified in the colon cancer samples, 11 are currently targets of FDA-approved drugs or drugs in clinical trials^55^, including APEX1, SERPINA1, and CASP7. More importantly, the NIPS method provides a novel insight into the understanding of the complex relationship between the occurrence of SAPs and disease at a view of structural protein interaction network. Some mutations are deleterious, primarily because these mutations rarely occur during evolution, disrupt interactions in the 3D protein structure, or induce changes in topology and signal transmission in the PPI network.

The AUC value of each of individual score (Fig. 2a) revealed that the I-score performed worse than the other scores, with an AUC value of 0.70. Although the sensitivity of the I-score was only 0.41, Its specificity was 0.99 when the I-score was used alone in the prediction, which implied that the deleterious SAPs identified using the I-score are likely to indeed be harmful. The low sensitivity likely reflects the low coverage of the I-score. Fortunately, with the rapid development and application of cross-linking mass spectrometry, the coverage of 3D structure of protein interactions is increasing rapidly, and a significant improvement in performance of the I-score would be expected in the near future.

In comparisons across multiple methods, NIPS and CHASM showed significantly better performances than the other methods. LogRE and SIFT, which use individual protein domains or protein sequences, displayed a lower accuracy. PolyPhen-2 performed slightly better than SIFT, though its strategy combines sequence and structural information. The poor accuracy of these three methods in predicting deleterious SAPs in cancer might reflect the lack of consideration of the specificity of the cancer genome in their algorithms. In contrast, the training systems of NIPS and CHASM use the known cancer-related genes and the frequency of missense mutations in the cancer somatic mutation database, respectively. The results suggest that specificity should be addressed to improve prediction accuracy. The performance of NIPS was similar to that of CHASM and showed higher overall accuracy and sensitivity but lower specificity. In CHASM, the model was trained using 49 features based on information on exon conservation, UniProt annotation and the frequency of missense mutations from a large-scale cancer genome project. In NIPS, only three feature scores, based on sequence conservation, protein interaction structure and interaction network topology, were employed. Moreover, NIPS provides the prioritization of deleterious SAPs and allows explanation of the results with respect to the 3D protein interaction or interaction network. Thus, NIPS could represent a good alternative and complementary method to existing methods for the prediction of deleterious SAPs. Additionally, this model can be extended to other diseases, but only if disease-specific training data are used.

The molecular dynamics simulations in this study showed that a deleterious SAP could alter the structure of the protein complex, thereby affecting the molecular function of the protein complex. A recent study of Yates *et al*.^22^ has demonstrated that the presence of a protein mutation might lead to derailment of the entire protein interaction network, directly resulting in the disease phenotype. In order to elucidate the dynamic impact of deleterious SAP in the network, the differential expression and modification profiles of all of downstream targets can be considered in the further study.

Recent studies involving the dynamic mathematic modelling of human tumour initiation and progression indicate that most somatic mutations observed in common tumours do not play any causal role, and only driver mutations are effectual^7,56–58^. Here we have shown the good performance of the NIPS algorithm for ranking deleterious SAPs in the cross-validations. We also identified 85 deleterious SAPs in the colorectal cancer cohort using NIPS, of which the accumulation of the top deleterious SAPs was significantly associated with a higher risk of prognosis. However, it should be noted that the follow-up wet-lab validations are essential for the novel driver SAPs predicted by *in silico* method before making a real application. Moreover, the association analysis between the top deleterious SAPs and prognosis was conducted in the TCGA colorectal cancer (CRC) samples only. Further studies in independent cohorts can be carried out by considering of clinical stages and subtypes, which will likely facilitate more effective prognostication efforts.

## Methods

### Training datasets

The deleterious SAPs identified by Gnad *et al*. were used as the positive training dataset^12^. According to this previous report, 2,682 somatic mutations were found in at least two tumour samples from the COSMIC database were defined as deleterious mutations. A total of 7,170 variants with a minor allele frequency of at least 0.25 in dbSNP (Build ID 135) were used as the negative training dataset, as relatively frequent mutations are unlikely to be deleterious^59^.

### Candidate somatic SAPs

In the CPTAC project, Zhang *et al*. identified 796 non-duplicated single amino acid variants (SAAVs, also known as SAPs) from 95 colorectal cancer samples via RNA-Seq and shotgun proteomics^30^. These SAPs were used in the application of the new method developed in the present study.

### PPI network and structural annotation

The high-quality HINT interactome was used as a background network to measure network topology^60^. The data in HINT were collected from BioGrid, DIP, HPRD, IntAct, iRefWeb, MINT, MIPS, and VisAnt^61–67^. Low-quality interactions were filtered and systematically and manually removed; thus, only confident physical interactions remained. The new interactions detected via cross-linking were also added. Self-interactions and duplicates were removed, leaving 5,585 edges with 3,280 proteins in the background PPI network.

As shown in Table 2, we collected experimentally validated protein interaction interfaces from four studies published since 2012, in which total protein interactions were profiled using cross-linking mass spectrometry^20,27–29^. To improve coverage, we added a three-dimensional (3D) protein interactome with structural resolution using INstruct^24^. INstruct employs iPfam and 3 did to identify the interface of two interacting proteins by mapping the proteins to known atomic-resolution 3D structures in the Protein Data Bank (PDB)^68–70^.

**Table 2:** Data sources for the human 3D protein interaction network.

Data Sources	Pairs	Proteins	Method
INstruct	4,292	2,623	Prediction
Herzog *et al*.^20^	195	81	Cross-linking
Chavez *et al*.^27^	146	128	Cross-linking
Kaake *et al*.^28^	16	15	Cross-linking
Liu *et al*.^29^	1,727	896	Cross-linking
Total	5,585	3,280	

### NIPS ranking scores

#### I-score (interface score)

We scanned the 3D network to determine whether a SAP was located at the interface of two interacting proteins. If so, the SAP received an I-score of 1; otherwise, the SAP received a score of 0. If a protein was not present in the 3D-network, then it received a score of 0. SAPs located at the interaction interface were considered likely deleterious SAPs.

#### T-score (topology score)

We first calculated the shortest path between each node (protein) in the background PPI network, and subsequently compared these paths with the positive dataset described above. According to the Cancer Gene Census database^71^, there are 451 cancer-related nodes in the network, which are defined as deleterious in cancer, whereas the other nodes are considered neutral. Next, we calculated the average length of the shortest path from each node to cancer-related nodes and neutral nodes.

1 $$Tscor{e}_{i}=\,\frac{Average\,{L}_{n}}{Average\,{L}_{c}}$$

where average ${L}_{n}$ is the average length of the shortest paths from node i to all neutral nodes, and average ${L}_{c}\,$is the average length of the shortest path from node i to all cancer nodes. The T-score reflects whether a node lies closer to a cancer-related node or to a neutral node in the network topology. Therefore, a node is more likely to be a potential deleterious node, and mutation of that node is likely deleterious if the node receives a higher T-score (above 1). Proteins that were not found in the network were defined as random nodes with the same average length of the shortest path to cancer nodes and neutral nodes; therefore, these proteins received a T-score of 1. We normalized the T-scores to between 0 and 1 and selected a 0.9 cutoff value via the ROC method using the R package “Daim”.

2 $$\text{Normalized}\,Tscor{e}_{i}\,=\,\frac{Tscor{e}_{i}-Tscor{e}_{min}}{Tscor{e}_{max}\,-\,Tscor{e}_{min}}$$

#### S-score (SIFT score)

SIFT first performs multiple sequence alignment of homologous proteins and identifies conserved protein residues based on the probability of each of the 19 amino acid changes being tolerated, relative to the most frequent residue. Less conserved protein changes are considered neutral, and more highly conserved protein changes are considered deleterious^3^. We obtained SIFT 5.1.1 from http://sift.bii.a-star.edu.sg, and used the UniProt database from EMBL (ftp://ftp.ebi.ac.uk/pub/databases/ fastafiles/uniprot/) as a reference sequence database, with a default cutoff score (0.05). SIFT depends on PSI-BLAST^72^; therefore, blast-2.2.26 was downloaded from ftp://ftp.ncbi.nlm.nih.gov/ blast/executables/. We defined the S-score as one minus the SIFT score; therefore, when the S-score is larger, the more likely the SAP will be deleterious.

#### Meta-score

We combined these three normalized scores into a weighted average score, referred to as the ‘meta-score,’ using the previously published Condel method^73^.

3 $${\rm{WAS}}=\frac{{\sum }_{i}{S}_{i}\,\ast \,{W}_{i}}{{\sum }_{i}{W}_{i}}\,$$

$${W}_{i}=1-{P}_{{n}_{i}}\,(if\,a\,mutation\,is\,predicted\,as\,deleterious\,with\,the\,{i}^{th}\,score)$$

$${W}_{i}=1-{P}_{{d}_{i}}\,(if\,a\,mutation\,is\,predicted\,as\,neutral\,with\,the\,{i}^{th}\,score)$$

${S}_{i}$ is the normalized score generated using the ${i}^{th}$ individual method, and ${W}_{i}$ is the corresponding weight of the given score. Based on the Condel score methodology, the weights are calculated on the basis of the probability of ne neutral (${P}_{{n}_{i}}$) or deleterious (${P}_{{d}_{i}}$) mutations with normalized scores higher than ${S}_{i}\,$in the training dataset, according to Gonzalez-Perez A *et al*.^73^. If a protein does not receive a score from an individual method, the corresponding weight is set to 0. The cutoff of the meta-score (0.9) was chosen based on the score distributions in the training dataset (Supplementary Fig. S1).

### Other tools for deleterious SAP prediction

#### PolyPhen-2

This software was downloaded from http://genetics.bwh.harvard.edu/pph2, and we followed the standard instructions for installation and operation.

#### LogRE

The software HMMER 3.0 (http://www.hmmer.org/) was used to align wild type and mutant protein sequences against Pfam protein domain models^14,74^. LogRE scores were then calculated using E-values from HMMER according to the strategy of LogRE^13^.

#### CHASM

This tool is a web-based application within CRAVAT (http://www.cravat.us) and is easy to apply online.

### Cross-validation

To validate our method, we conducted 10-fold cross-validations using the training datasets. Then, we drew ROC curves to evaluate the S-scores, T-scores, I-scores, and meta-scores and calculated AUC values to compare the performance of the methods.

### Molecular dynamics simulation

We performed molecular dynamics simulations (50 ns) in AMBER12 for the wild-type and mutant protein sequences to validate the influence of each mutation on protein binding affinity^75^. The binding free energy in GB (Generalized Born) mode can indicate the binding affinity of proteins^76,77^. We used MMPBSA in AMBER12 to calculate the binding free energy in GB mode for both types^78^.

### Survival analysis

Clinical prognosis information was available for a total of 86 patients among the 95 colorectal cancer samples in CPTAC^30^. We summed the meta-scores of the top 30 deleterious SAPs predicted using NIPS, and divided the samples into two groups (above or below the median). Then, survival analysis and Cox proportional hazards regression were conducted using the *survival* package in R.

## Electronic supplementary material

Supplementary Information
