# Supplementary material for: NIPS, a 3D network-integrated predictor of deleterious protein SAPs, and its application in cancer prognosis
Source: Sci Rep. 2018 Apr 16;8:6021. doi: 10.1038/s41598-018-24286-2 (PMC5902451; doi:10.1038/s41598-018-24286-2)
Supplement: Supplementary file 1 — Supplementary Information [file 41598_2018_24286_MOESM1_ESM.pdf]

NIPS, a 3D network-integrated predictor of deleterious protein SAPs, and its application in cancer prognosis

Bo Wang[1], Jing Li[1], Xi Cheng[1], Qiao Zhou[1], Jingxu Yang[1], Menghuan Zhang[1], Haifeng Chen[1], Yixue Li[1,2], Jing Li[1*]

[1] Department of Bioinformatics and Biostatistics, School of Life Sciences and Biotechnology, Shanghai Jiao Tong University, Shanghai, China

[2] Key Laboratory of Computational Biology, CAS-MPG Partner Institute for Computational Biology, Shanghai Institutes for Biological Sciences, Chinese Academy of Sciences, Shanghai, China

* jing.li@sjtu.edu.cn

Corresponding author: **Jing Li**

Supplementary Figures and Tables

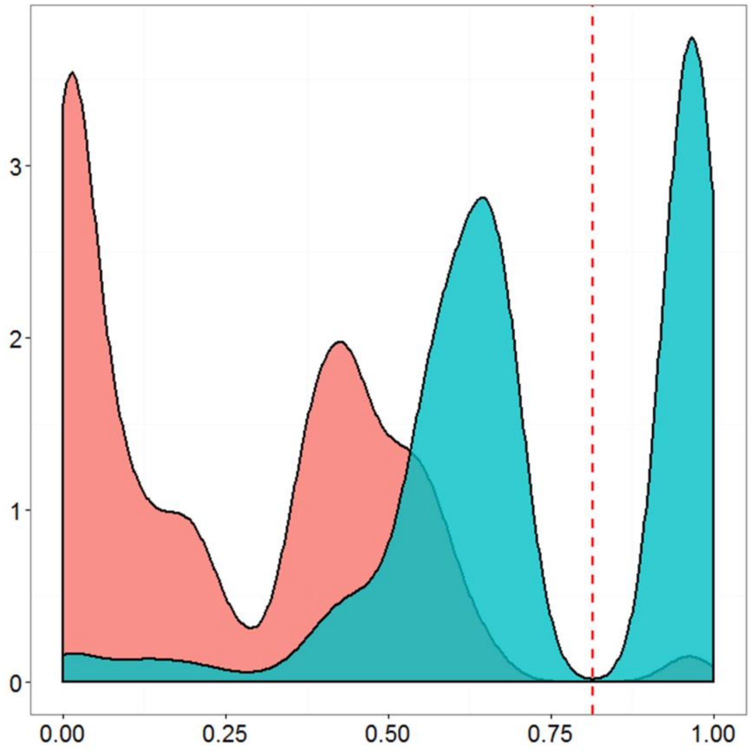

Supplementary Figure S1. The distributions of the Meta-scores of the positive dataset (blue, deleterious) and the negative dataset (blue, neutral).

Supplementary Table S1. The list of deleterious mutations in human colorectal cancer samples that were identified by NIPS

**Rank**	**Protein**	**SAP**	**Meta Score**	**S-Score**	**T-score**	**I-Score**	**rsid**	**COSMIC**	**Publication support (PMID)**
1	CYBA	Y72H	1	1	0.89	1	rs4673		21640156; 15860042; 15078863
2	SEC23B	H489Q	0.99	0.99	0.89	1	rs2273526		29031773
3	PSMA7	V102I	0.99	0.15	0.88	1			
4	CYBA	V174A	0.99	0.02	0.89	1	rs1049254		
5	MYH9	L46F	0.98	1	0.94	1	rs147122501		24130771
6	CYFIP1	G820S	0.98	0.26	0.89	1	rs7170637		
7	GSN	T576M	0.98	0.98	0.95	1	rs76463933		
8	SRSF3	N20S	0.98	0.27	0.89	1		COSM298507	22810696
9	HLA-A	G80R	0.98	0.99	0.95	1			
10	HSP90AB1	R400C	0.98	1	0.95	1			
11	GSPT1	G92C	0.98	1	0.93	1	rs11544193		
12	CTSG	N125S	0.98	0.24	0.89	1	rs45567233		
13	STAT1	P538L	0.98	0.98	0.96	1	rs1803838	COSM209252	

14	CTSS	R205H	0.97	0.99	0.9	1	rs138263900		
15	SEC23A	L211V	0.97	0.63	0.88	1	rs8018720		
16	COPB2	Y194C	0.97	0.98	0.92	1			
17	ACTC1	T151A	0.97	1	0.92	1			
18	HEXA	I436V	0.97	0.5	0.89	1	rs1800431		
19	CTNNB1	W383G	0.97	0.47	0.97	1		COSM290306	20579941;27760322; 26071483;22653804; 22810696; 28930697
20	APEX1	D148E	0.97	0	0.95	1	rs1130409		
21	CASP7	D255E	0.97	0.07	0.94	1	rs2227310		
22	SERPINA1	E366K	0.97	1	0.92	1	rs28929474		
23	ETFA	T171I	0.97	0.99	0.91	1	rs1801591		
24	PSMB3	M34L	0.97	0.89	0.89	1	rs4907		
25	KRAS	G12D	0.97	1	0.91	1	rs121913529	COSM14209	25705018; 27624879; 26567140; 21996748
26	VCL	R230C	0.97	1	0.91	1	rs139312390		
27	KRAS	G13D	0.97	1	0.91	1	rs112445441	COSM531	22734028; 26371285 19246520; 15235871
28	MCM4	N618D	0.97	1	0.91	1			
29	SOD1	D91A	0.97	0.84	0.83	1	rs80265967		
30	KRT8	R341H	0.97	0.97	0.95	1	rs57422427		16729313; 25963979; 22412904
31	SERPINA1	E400D	0.96	0	0.92	1	rs1303	COSM147838	
32	COL4A2	V1399I	0.96	0.06	0.9	1	rs45520539		
33	CFL1	K95Q	0.96	0.28	0.94	1		COSM298895	22810696
34	HLA-A	R138Q	0.96	0.69	0.95	1			
35	HLA-DQB1	D89V	0.96	0	0.91	1	rs1071637		
36	SERPINA1	E288V	0.96	0.94	0.92	1	rs17580		
37	ITGB2	Q354H	0.96	NA	0.92	1	rs235330		
38	COL4A2	R517K	0.96	0	0.9	1	rs7990383		
39	SERPINA1	V237A	0.96	0.2	0.92	1	rs6647		
40	MCM6	V376I	0.96	0.97	0.9	1		COSM204646	22895193
41	JUP	V456I	0.96	0.26	0.94	1	rs78437817		
42	SEC23B	V426I	0.96	0.52	0.89	1	rs41309927		
43	XRCC5	A550S	0.96	0.35	0.93	1	rs35408277		
44	USH1C	E519D	0.96	0.59	0.89	1	rs1064074		
45	SCFD1	V143I	0.96	0	0.9	1			
46	SEC23B	P433L	0.96	0.69	0.89	1	rs17807673		
47	CCT5	I362M	0.96	0.95	0.93	1	rs141675330		
48	HLA-DRB3	L67V	0.96	0	0.92	1			
49	TF	P589S	0.96	0.87	0.94	1	rs1049296		

50	NCF1	G99S	0.96	NA	0.92	1	rs17856077		
51	AURKB	M298T	0.96	0.46	0.94	1	rs1059476		
52	LMNB1	D155E	0.96	0	0.92	1		COSM203285	
53	USP5	R415W	0.96	0.98	0.91	1			
54	HLA-DRB1	V114A	0.95	0.52	0.93	1	rs17424145		
55	DSG2	R773K	0.95	0.86	0.92	1	rs2278792		
56	SCFD1	R549H	0.95	0.92	0.9	1	rs150891719	COSM184976	
57	ACTG1	M153V	0.95	0.63	0.92	1			
58	U2AF2	N192D	0.95	0.46	0.92	1			
59	ARL2	V141A	0.95	0.31	0.9	1	rs664226	COSM1132962	
60	NCKAP1	Q774R	0.95	0.65	0.92	1			
61	RAB11A	R33Q	0.95	0.87	0.91	1		COSM263200	22810696; 24755471
62	ARHGAP1	I261T	0.95	0.62	0.93	1			
63	TMED10	D125Y	0.95	0.93	0.91	1		COSM176453	
64	GSTO1	A140D	0.95	0.38	0.91	1	rs4925	COSM147037	23888047; 22440634;22885077; 18941778; 18400112
65	HLA-DQA1	Q198K	0.95	0.4	0.93	1	rs1129957		
66	CSE1L	A276T	0.95	0.42	0.92	1	rs149344844	COSM189825	
67	ITGA6	A380T	0.95	0.93	0.91	1	rs11895564		
68	SRPK1	R649Q	0.95	0.59	0.92	1	rs35124200		
69	PSMB1	K77E	0.95	0.19	0.9	1			
70	MYH9	E334D	0.95	0.52	0.94	1			
71	KRAS	G12S	0.95	0.85	0.91	1	rs121913530	COSM517	27863474;27252416; 25245423;23455880; 29348108;28621683
72	HSPD1	I318V	0.95	0.81	0.92	1			
73	HLA-DQA1	M99V	0.95	0.57	0.93	1	rs1064944		
74	PTGES3	S39G	0.95	0.58	0.94	1		COSM192056	
75	ARHGAP1	L263F	0.95	0.76	0.93	1	rs144801476		
76	POLR2A	G50D	0.95	0.76	0.93	1			
77	PIGR	G365S	0.94	0.8	0.9	1	rs2275531		
78	TIMP1	T171M	0.94	0.83	0.9	1			
79	ANP32A	S104N	0.94	0.4	0.9	1		COSM194539	
80	VWF	T1381A	0.94	0.47	0.9	1	rs216311		
81	LASP1	V44I	0.94	0.54	0.91	1			
82	HEXB	I207V	0.94	0.69	0.91	1	rs10805890		
83	MTTP	H297Q	0.94	0.52	0.91	1	rs2306985		
84	SCFD1	K63R	0.94	0.66	0.9	1	rs229150		
85	DNAJA1	S49A	0.94	0.62	0.91	1		COSM260514	22810696
